# Preparation and Characterization of New Environmentally Friendly Starch-Cellulose Materials Modified with Casein or Gelatin for Agricultural Applications

**DOI:** 10.3390/ma12101684

**Published:** 2019-05-23

**Authors:** Mirosława Prochoń, Anna Marzec, Bolesław Szadkowski

**Affiliations:** Institute of Polymer and Dye Technology, Faculty of Chemistry, Lodz University of Technology, Stefanowskiego 12/16, 90-924 Lodz, Poland; anna.marzec@p.lodz.pl (A.M.); boleslaw.szadkowski@edu.p.lodz.pl (B.S.)

**Keywords:** starch, casein, cellulose, biodegradation, composites

## Abstract

The purpose of this work was to prepare new biodegradable starch-cellulose composites, with starch, using casein and gelatin as natural nutrients. The physico-chemical properties of the starch films and cellulose fabrics with starch coatings were studied by Fourier transformation infrared analysis, laser confocal scanning microscopy (LCSM), scanning electron microscopy (SEM), UV-Vis spectroscopy, swelling tests, mechanical tests, thermal analysis thermogravimetric analysis (TGA), and differential scanning calorimetry (DSC). The susceptibility of the starch films to biodegradation was investigated, together with their resistance to thermo-oxidative aging. As a result of the formation of the starch films, both the casein and gelatin macromolecules were able to interact directly with the starch matrix and the fractions of unbranched amylose and branched amylopectin it contained. This interaction was visible as changes in the absorption bands of the polar groups, as revealed by infrared analysis. Spectral analysis of the cellulose fabrics coated with starch films suggests that hydrogen bridges formed between the micelles of long cellulose filaments and the micro and macro-fibers of the starch pectins. An applicative test revealed that when used as a covering for bean cultivation the cellulose-starch composites act as a fertilizing component, contributing to significantly improved growth of *Phaseolus vulgaris* in comparison to the use of unmodified cellulose.

## 1. Introduction

The treatment of polymer waste has been recognized in recent decades as a major cause of environmental pollution. In agriculture, synthetic materials such as polymer mulches are applied to increase crop yields as well as to control weeds and pests. However, synthetic materials have a negative impact on the environment, due to their long degradation time. Therefore, there is increasing interest in the design and production of novel biodegradable composites from renewable resources [1,2,3,4,5,6].

Most films on the world market produced for agricultural applications are made of linear LLDPE (Linear Low-Density Polyethylene). Another important material is the EVA/EBA (ethylene-vinyl acetate EVA/ethylene-co-n-butyl acrylate EBA) copolymer, a combination of ethylene and vinyl acetate copolymer with built-in ethylene chains modified by butyl acrylate. The EVA/EBA copolymer is used to produce technical textiles, including garden foils. The copolymer is characterized by very good flexibility as well as chemical and thermal stability, making it a very desirable material [7,8,9]. Agro-nonwoven fabric can be made of polypropylene (PP), nylon, polyester fibers, etc. It has an interwoven irregular structure with impressed grooves, thanks to which it is very durable and provides good surface protection against weeds. Nonwovens are produced using the technology of air needling, while synthetic fabrics are made by extrusion through a slot nozzle, stretching and extrusion with a free blow. Unlike foil, agro-textiles are perfectly permeable to water and minerals, and do not decay. As a result of their high strength, durability and other properties, synthetic fibers are widely used in the agro-tech sector. However, due not only to the toxicity of the materials used in the production of the substrates but also to the harmful substances generated in the course of chemical processing, films and coverings which are biodegradable and environmentally friendly are becoming more and more popular [10].

Although artificial fibers (such as polyolefins) are preferred to natural fibers for use in agro-textiles, due mainly to their better price/price ratio, low weight, high strength and long life, natural fibers can be used in some specific areas, where properties such as high moisture retention, wet strength, biodegradability are required [11]. Jute, wool, coconut, sisal, flax and hemp fibers are examples of natural fibers [12]. Agro-fabrics based on natural fibers not only serve a specific purpose, but also degrade after a year and act as natural fertilizers. Natural nonwovens may be used to produce special blankets containing seeds for further germination [13]. Researchers [14] have compared the impact of natural coverings and synthetic mulches on the growth of fruit trees. Four-year studies have shown that it is better to use linseed agro-textile than a synthetic foil or tree bark, with simple soil reclamation used as the control. Currently available biodegradable agricultural films may also contain starch, PLA (polylactic acid) polylactide, natural co-polyesters or cellulose. Disadvantages include the price (for instance, of PLA), but above all the difficulty of processing the material. Nevertheless, the potential of natural agro-textiles and artificial agro-textiles is huge, and at the same time there is growing demand.

Starch-type biopolymers, casein, gelatin, etc., seem promising materials for use in natural fibers. One such natural polymer is starch, which has many unique properties. Starch is a polysaccharide consisting of amylose and amylopectin. The ability to gelatinize is one of the most important properties of starch. In this process, the intermolecular bonds of starch molecules are broken down in the presence of water and heat. Many applications of starch rely on its ability to gelatinize and on its solubility or swelling power [15,16,17,18,19,20]. The advantages of starch-based materials include low price, biodegradability and renewability. Biodegradable starch-based materials are already used in food packaging.

The aim of the present study was to obtain and characterize starchy films and cellulose-starch composites (agro-textiles coated with the starch films). The starchy films were made by chemical modification of starch using gelatin or casein as the binder and coating–forming factors. Casein was chosen due to its film–forming properties and broad range of applications in food, textiles, adhesives, etc. and gelatin due to its gelling properties and the possibility of sol in the gel, as well as for its wide applications in the food industry, photography and industry pharmaceuticals [5,6,7,21,22,23,24,25,26,27,28,29,30]. Next, cellulose-starch composites were obtained by modifying a commercially available cellulose coating using the starch films. The physico-chemical properties of the materials produced were analyzed and the probable reaction mechanism through which the starch materials formed was determined. The final composites are intended for use in the agricultural sector (gardening, floriculture, development of urban areas, embankments etc.). To the best knowledge of the authors, there has been no previous research on how the application of gelatin and casein affects plant growth and adhesion to agro-fabrics. The agro-fabric produced is biodegradable and can be composted.

## 2. Materials and Methods

### 2.1. Materials

Starch SUPERIOR was obtained from Trzemeszno, Poland. Casein and gelatin were produced by Chemico, Białe Błota, Poland. Cellulose fabric was sourced from MATIMPEX PPHU, Lodz, Poland. Ammonia was obtained from Chempur, Piekary Śląskie, Poland. Glycerin was supplied by Chemico, Białe Błota, Poland.

### 2.2. Preparation of Starch Films and Cellulose-Starch Composites

Mixtures were prepared according to the formulas presented in Table 1.

**Figure 1:**
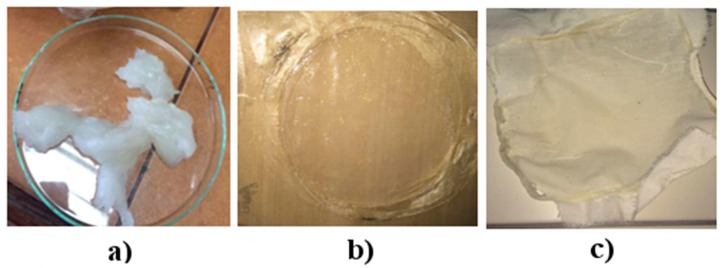
Starch mixture before pressing (**a**), starch film (**b**), cellulose-starch composites (**c**).

The process of preparing the mixture was based on gelatinization (T = 65 °C, t = 2.5 h) of the respective weight fraction of starch (Table 1), to which 10 or 25 parts by weight of glycerol were introduced. The mixture was stirred at 80 °C until a suitable consistency was obtained. A corresponding proportion by weight of casein or gelatin was then introduced (Figure 1a). The reaction mixture was divided into two parts. Starch films were made from one, and starch-cellulose composites from the other.

Starch films with a thickness of 1 mm were obtained by pressing the starch mixture on a hydraulic press (T = 100 °C, p = 20 Ba) (Figure 1b). The starch and cellulose composites were made using the coating method. The starch films were applied to the cotton fabric and stretched using a paint roller. A hydraulic press (Skamet 54436, SKAMET, Skarżysko-Kamienna, Poland) was used for thermal stabilization of the starch-casein and starch-gelatin composition on cotton fabric. This process was conducted at a temperature of 100 °C and at a pressure of 150 bars for 5 min. As a result, cellulose-starch composites were obtained (Figure 1c).

Appropriate forms were cut from the fabric-coated compositions, in accordance with the applicable standards for the various test techniques. Immediately after their preparation, the starch mixtures were incubated under conditions of about 7 °C or directly formed in a hydraulic press.

Importantly, during the production of the composites from natural raw materials the appearance of fungi or mold was noted on their surfaces. The appearance of microorganisms on the polymeric material was the result of storage for 3 days under standard room conditions and will be discussed in Section 3.1.4.

### 2.3. Properties of Starch Film and Cellulose-Starch Composites

#### 2.3.1. Mechanical Properties

The mechanical properties of the prepared composites were tested using a Zwick universal testing machine model 1435 (DEGUMA-SCHÜTZ GmbH, Geisa, Germany) according to the PN-ISO 37:1998 standard with a preliminary force of 0.1 N and strain rate of 200 mm min^−1^. The tensile strength (TSb) and percentage elongation at break (Eb) were determined.

Hardness measurements were performed according to the PN-71/C-04238 standard. A SHORE C digital hardness meter (Zwick GmbH&Co, Ulm, Germany, pressure 50 N) was used to test the coated fabrics and a SHORE A (pressure 12.5 N) to test the films (Zwick GmbH&Co, Ulm, Germany). Measurements are made in accordance with the applicable standards: DIN EN ISO 868, DIN 53505, ASTM D 2240, ISO 7619, NFT 51-174, BS903 PART A 26. Hardness was determined before and after aging.

#### 2.3.2. Equilibrium Swelling

The swelling index was determined using the equilibrium swelling method. This test was carried out according to the PN-ISO 1817:2011/Ap1:2002 standard. Specimens with an average mass of 30–40 mg were immersed in pure water. Measurements were carried out at room temperature (25 °C) for 2 days (48 h). After this time, the samples were removed from the immersion liquid to determine their weight change. The samples were washed with diethyl ether, then dried on filter paper and weighed. Next, the samples were dried in air for 7 days. They were then subjected to drying at a temperature of 50 °C for 96 h. Their weight stability was then tested by additional measurements. The swelling ratio was calculated according to the following Equation (1):

Qw = (m − ms)/ms,(1)

where m [g] and ms [g] are the mean weights of the analyzed samples before and after swelling in the immersion liquid, respectively.

#### 2.3.3. Thermo-Oxidative Aging Resistance

Thermo-oxidative aging resistance was determined according to the PN-88/C-04207 standard. Samples were placed in a drier with an air circulation system (Binder 07-30141, Binder Sweden KB, Stockholm, Sweden). The samples were left for 7 days at a temperature of 70 °C. The tensile strength (TSb) and percentage elongation at break (Eb) were determined again. Based on TSb and Eb values, the ageing coefficient (A) was calculated from the Equation (2):

(2) $$A=\frac{{\mathrm{TS}}_{b2}\times E_{b2}}{{\mathrm{TS}}_{b1}\times E_{b1}}$$

where: TS_b2_—tensile strength [MPa] after thermal-oxidative aging; E_b2_—elongation at break [%] after thermal-oxidative aging; TS_b1_—tensile strength [MPa] before thermal-oxidative aging; E_b1_—elongation at break [%] before thermal-oxidative aging.

#### 2.3.4. Fourier Transformation Infrared Spectrometer and Microscopic Analysis

The samples were characterized by means of Fourier Transformation Infrared (FTIR) spectroscopy using a Nicolet 6700 (Thermo Scientific, Waltham, MA, USA) equipped with a Platinum ATR single reflection diamond, USA. Fifty scans were obtained at a resolution of 10 cm^−1^ from 4000 to 600 cm^−1^ wavenumber, with a set resolution of 8 cm^−1^ and a scan number of 64. A confocal Keyence Laser Scanning Confocal Microscopy (LSCM, KEYENCE NV/SA, Mechelen, Belgium) system was used to acquire optical images in 3D with high resolution lenses: 220 μm × 50 μm; 220 μm × 100 μm and 2500 μm × 500 μm (Mechelen, Belgium).

#### 2.3.5. Color Characteristics

Color changes in the composites after incubation in universal soil were measured suing a CM-3600d UV-VIS spectrophotometer (Konica Minolta, Tokyo, Japan). The total color difference (∆E*) was evaluated from the range of changes, with higher ∆E* values indicating greater observed changes.

#### 2.3.6. Soil Tests

Soil aging tests were carried out according to PN-EN ISO 846. Fittings in the form of paddles were placed in universal soil in a climatic chamber (MEMMERT type HPP 108, Memmert GmbH + Co. KG, Schwabach, Germany) for 15 days at 30 °C with relative humidity of air WWP = 80%.

#### 2.3.7. Study of Susceptibility to Biodegradation of Starch Films and Cellulose-Starch Composites

As a result of storage at room temperature (3 days), mold and similar microorganisms appeared on the surfaces of the starch films. The Institute of Microbiology at Lodz University of Technology investigated whether such fungi would appear under similar conditions on a composite material made of biopolymers, such as starch, gelatin or casein. Mold fungi chosen in accordance with PN-EN ISO 846 were used as test organisms. A mixture of the following mold fungus species was used: Aspergillus niger, Penicillum ochrochloron, Aspergillus terreus, Scopularopsis brevicaulis, Fuzarium, Rhizoctonia, Claviceps and Stachybotrys and Chaetomium globusum. Studies were also conducted on dermatophytes from the species Trichophyton mentagrophytes and actinomycetes of the genus Streptomyces. The strains were obtained from the Collection of Pure Cultures for Industrial Microbiology at the Institute of Fermentation and Microbiology Technology, PL, LOCK 105 [31].

#### 2.3.8. Optical and SEM Microscopic Analysis

An optical microscope with digital imaging was used for 2D measurements in real time and for 3D imaging of surface topography (Keyence, Mechelen, Belgium).

The surfaces of the obtained composites were assessed by SEM with a Bal-Tec SCD050 sputter coater and LEO 1530 produced by Bal-Tec (Raith GmbH Dortmund, Germany).

#### 2.3.9. Thermal Analysis

The thermal stability of the material was determined using a Mettler Toledo TGA/DSC (thermogravimetry/differential scanning calorimetry) analyzer (Mettler Toledo, Greifensee, Switzerland) calibrated using the standard pattern (indium, zinc). The samples were heated at a heating rate of 10 °C min^−1^ in a stream of inert gas (argon) with a flow rate of 60 cm^3^ min^−1^ in a temperature range of 25–800 °C. The heat effect and the range of the glass transition temperature were determined by differential scanning calorimetry (DSC) in the temperature range of −80 to 150 °C, with a heating rate of 10 °C min^−1^.

#### 2.3.10. Sowing Beans on Cellulose-Starch Composites and Composting Tests

The cellulose-starch composites were placed in containers containing soil with pH 5.5–6. Dwarf beans (Phaseolus vulgaris, PlantiCo, Stare Babice, Poland) were planted in the soil. Seeds were also planted in a container with no composite, as a control.

Composting tests were conducted in a climatic chamber with a humidity of 80% and a temperature of 30 °C in periods of 1 month, 3 months, half a year and 1.5 years. During the measurements, the composites were mechanically mixed and measured for the loss of mass. Structural changes in the composites were estimated organoleptically.

## 3. Results and Discussion

Analysis of the results was divided into two parts, the first relating to the properties of the starch films and the second focusing on the properties and possible applications of the cellulose fabrics covered with starch film.

### 3.1. Starch Films

#### 3.1.1. Fourier Transformation Infrared Spectrometry

**Figure 2:**
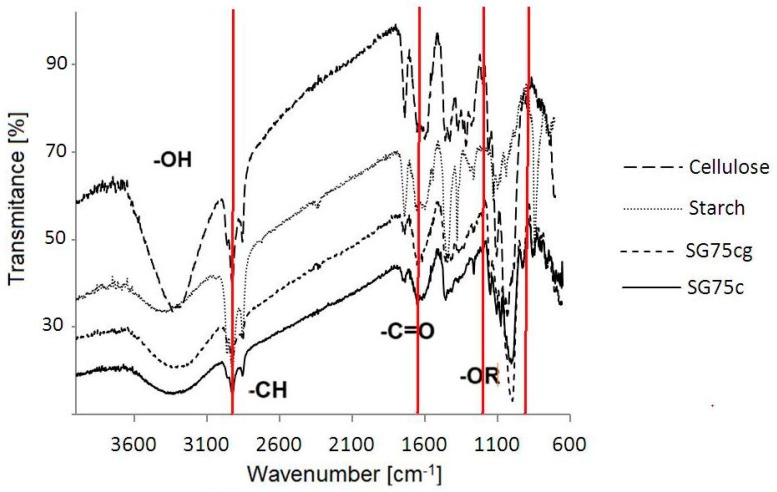
FTIR spectra of cellulose and starch as well as selected starch films containing casein (SG75c) or casein and gelatin (SG75cg).

Interactions between the starch matrix and other components were evidenced by changes in the absorption bands on the transmission spectra obtained by FTIR (Figure 2). The bands from the hydroxyl groups are visible in the range 3100–3600 cm^−1^, and water absorption is higher for the SG75cg film than for SG75c, due to the gelatinizing properties of gelatin and greater hydrophilicity of the gelatin system. The OH groups are most visible in the cellulose spectrum. We therefore had the idea of coating cellulose with a starch-casein or starch-gelatin mixture to increase the hydrophilicity of the composite intended for the cultivation of plants. This would ensure greater water absorption and better maintenance of the plants during periods of drought.

**Figure 3:**
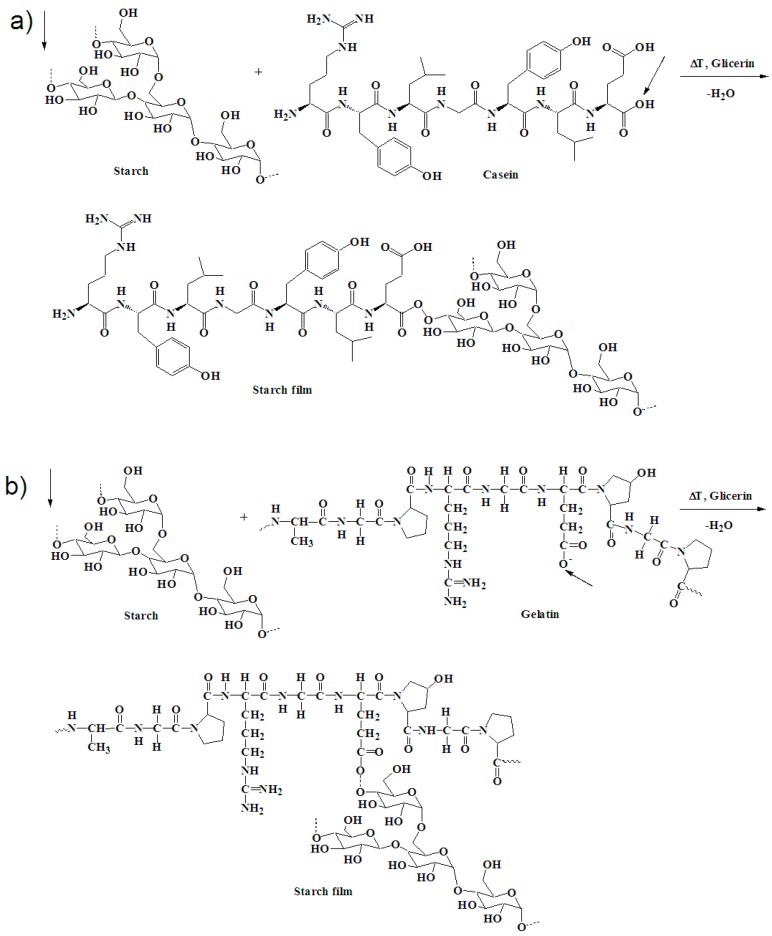
Probable interactions between starch and casein macromolecules (**a**) and between starch and gelatin (**b**).

The FTIR spectrum of the sample containing casein (SG75c) and casein/gelatin (SG75cg) shows the presence of –CH bonds. On the spectrum of both cellulose and starch, much more intense bands in the range of 3000–2800 cm^−1^ of the stretching vibrations of the –CH groups are visible, which, due to the introduction of either casein or the casein system with gelatin, were significantly shortened. This may indicate the creation of a common spatial network between the components of the composition during the cross-linking processes. The buildup of C=O bonds in the casein and gelatin sample (SG75cg) caused an increase in absorption band intensity in the range of 1500–1700 cm^−1^. The presence of bands attributed to the carbonyl and hydroxyl groups may be indicative of possible interactions in the lattice network between casein and gelatin macromolecules versus starch macromolecules. The starch films produced by thermal crosslinking in the presence of a plasticizer (glycerol) formed stable joints at elevated temperatures. New –OR groups were created at wavenumbers 1700 and 1100 cm^−1^, as shown in Figure 3a,b. The highest intensity of vibrations in the range from 1200 to 900 cm^−1^ was observed for the sample containing both casein and gelatin (SG75cg), as indicated by the occurrence of C–O bonding [32,33,34,35].

#### 3.1.2. Hardness and Equilibrium Swelling

Hardness (H) increased with increasing starch content relative to the glycerol plasticizer in the composites SG75, SG80 and SG85 (Table 2). The stiffness of the presented polymeric materials rises. The introduction of gelatin results in higher plasticity than the addition of the casein biopolymer, regardless of the starch and plasticizer used.

The results in Table 2 reveal that the introduction of casein or gelatin as modifiers in starch films leads to an increase in the infiltration of samples in water (Qw). As the proportion of plasticizer decreases, the penetration of water is limited to the structures of the shell composites. This may increase the spore properties of the starch substrates created, but also affects the possibility of applying additional fertilizing components to accelerate the growth of plants.

It can be also seen that samples containing gelatin and casein exhibited the most significant changes in terms of the hardness parameter after soil tests (∆H). The introduction of gelatin and starch promotes the biodegradation process, as evidenced by greater changes in parameter ∆H as well as by composting tests. A larger proportion of plasticizer in starch films provides greater protection against color changes under a half-dozen different external factors, which is indicated by the dE*ab parameter. In addition, the SG75c and SG75cg samples may exhibit greater color protection, for example against UV radiation.

#### 3.1.3. Mechanical Properties

The application of gelatin and casein as modifiers resulted in an increase in the hardness of starch films containing gelatin (Table 2). Due to the presence in gelatin of amino acids (such as proline, hydroxyproline or glycerin), the stiffness and hardness of the SG75g composite increased compared to the SG75cg sample. Casein contains phosphorous and glycoprotein, as well as incorporated sugar and phosphate residues, which are more compatible with gelatin than starch, which is a polysaccharide. After the introduction of gelatin into the structure of the composite, there was a slight increase in the hydrophilic properties of the material. On the other hand, the addition of casein slightly reduced the absorption of impurities into the polycarbonate structures. Casein is characterized by the increased presence of dissociated acidic groups over alkaline. This makes fractions in a pH 6.6–7 environment capable of forming ionic and hydrophobic bonds.

#### 3.1.4. Susceptibility to Biodegradation of Starch Films

**Figure 4:**
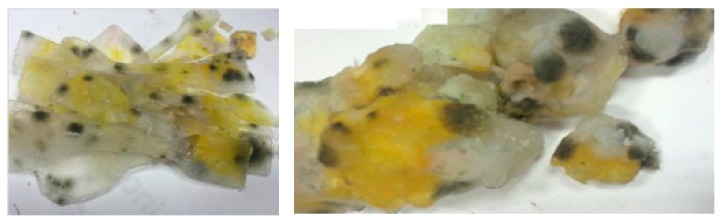
Samples of starch films undergoing biodegradation.

Non-crosslinked samples of the starch films were incubated at room temperature. This led to significant surface changes. Before thermal crosslinking, the samples the films were stored at room temperature for 3 days, not stored according to recommended practice in a thermal chamber at below 10 °C. This resulted in the formation of yellow-colored efflorescence as well as clusters of mold and fungi on their surfaces. Microbiological analysis carried out by the Microbiology Institute at Lodz University of Technology revealed that the surfaces were covered with dark green mycotoxin forms (Figure 4).

The toxins were produced by fungi (mold) from the genera Aspergillus, Penicillium, Fusarium, Rhizoctonia, Claviceps and Stachybotrys. The optimal temperature at which mycotoxins form is between 20–25 °C. Mycotoxins include, among others, aflatoxins, ochratoxins (including ochratoxin A), patulin and aspergillose acid. For example, yellow, red and green blooms may suggest a type of Aspergillus, brown Cladosporium, black and gray Aspergillus niger, Alternaria, Rhizopus or Mucor [31].

### 3.2. Cellulose-Starch Composites

#### 3.2.1. Mechanical Properties

**Figure 5:**
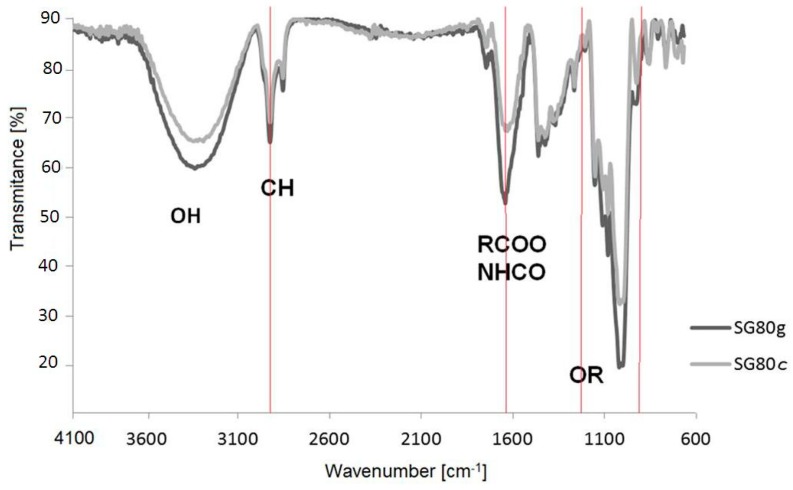
FTIR spectra of cellulose-starch composites.

**Figure 6:**
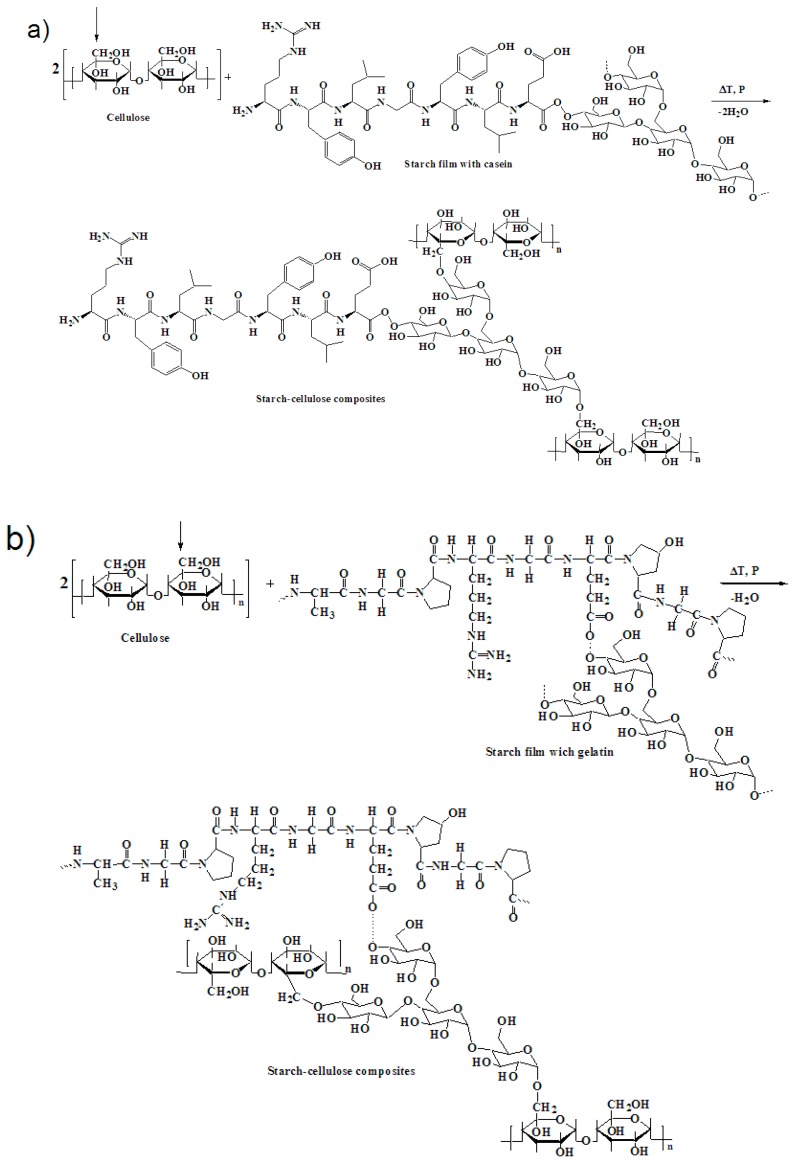
Proposed mechanism of interactions between cellulose and the starch-casein film (**a**) or starch-gelatin film (**b**) leading to the production of cellulose-starch composites.

Figure 5 shows FTIR spectra for the cellulose-starch composites, recorded in the range of 500–4000 cm^−1^. The main differences seem to be in the fingerprint region at 3600–3200, 1650 cm^−1^ and 1200 cm^−1^. The FTIR spectra clearly indicate possible interactions occurring in the polysaccharide chains between starch and cellulose macromolecules (reaction mechanisms Figure 6a,b).

The FTIR spectra of these samples show a strong absorption peak for the O–H stretching band centered around 3400 cm^−1^ and a minor C–H stretching band at 2940 cm^−1^. In these regions, the absorbance intensity recorded for the sample containing casein was higher compared to the sample with gelatin. This is connected with higher equilibrium swelling in the sample modified by casein (Figure 5). Typical peaks for symmetrical R–COO– groups and amide bands appear at 1630 cm^−1^ and 1510 cm^−1^, respectively. The lower absorption of the sample modified by gelatin at 1630 cm^−1^ may be associated with the participation of these groups in the formation of bonds between the components of the mixture. The formation of a larger number of polar groups, e.g., –CO or –OR, as can be seen in the FTIR spectra at a wavenumber of 1630 cm^−1^, suggests the creation of new connections, such as hydrogen bonds, ionic interactions, weak-range interactions, etc. between the starch-cellulose macromolecules and gelatin macromolecules (Figure 6b). The visible interactions are mainly derived from the branched amylopectin fractions in starch, which dissolve much more readily in cold water than unbranched starch amylose fractions and react with the filaments of cellulose fibers to form starch-cellulosic composites (Figure 6a,b). There was a significantly smaller amylose fraction in the sample with 14–27% starch compared to the amylopectin fraction, which results from the botanical origin of the starch itself. The amylopectin fractions are more branched, with high molecular weight, and form sol as a result of starch swelling [36,37]. This led to improved mechanical strength in samples modified by the addition of gelatin. The FTIR spectra of the sample with casein show a stronger absorption peak for the skeletal mode vibration of the glycoside linkage –OR (900–950 cm^−1^) [38,39,40].

#### 3.2.2. Equilibrium Swelling

**Figure 7:**
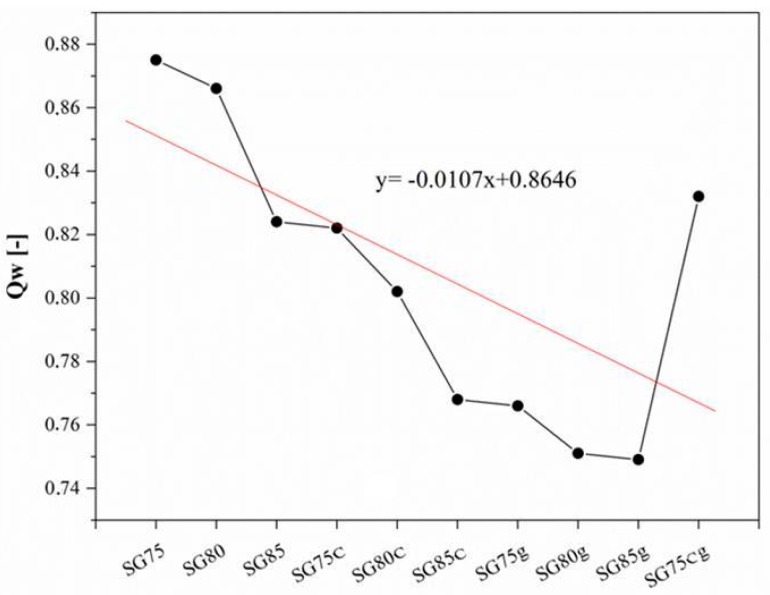
Effect of starch and glycerin content and of the addition of casein or gelatin as a modifier on equilibrium swelling in cellulose-starch composites.

Based on the results of equilibrium swelling measurements, the swelling ratio of the studied composites decreased with increasing starch content (Figure 7). A reduction in water absorption was observed in the analysis of shell films (Table 2) as the percentage of starch was increased. Specimen SG75 had the highest value for the swelling ratio, which is related to its also having the lowest hardness and tensile strength. The swelling ratio may also be connected with the crosslink density value of the sample. The results show that the crosslink density of the composites decreased with increasing casein content. This is an effect of the higher tensile strength of the samples containing a gelatin modifier.

The composites with greater cross-link density appear to have more hydrogen bonds between their individual components, such as starch, gelatin and the glycerin system (Figure 3 and Figure 6). This is reflected in their mechanical properties, such as tensile strength. The degree of cross-linking was noticeably better in those composites than for the hybrid systems, for example starch-gelatin. The regression curve correlates the value of the expected variable resulting from another variable. Variable factors include the introduction of biopolymers, casein and gelatin, into the starch matrix, as well as the varying proportion of glycerol plasticizer, from 15 to 25%. There is a linear change in the value of the equilibrium swelling parameter, which is driven by the decrease in the proportion of glycerol plasticizer together with the smaller share of glycerin (SG75, SG80, SG85). Equilibrium swelling Qw decreases linearly as a result of the introduction of casein (SG75c, SG80c, SG85c) and gelatin (SG75g, SG80g, SG85). However, the introduction of both casein and gelatin into the starch structure influenced Qw growth and the formation of a non-linear regression curve.

#### 3.2.3. Mechanical Properties

**Figure 8:**
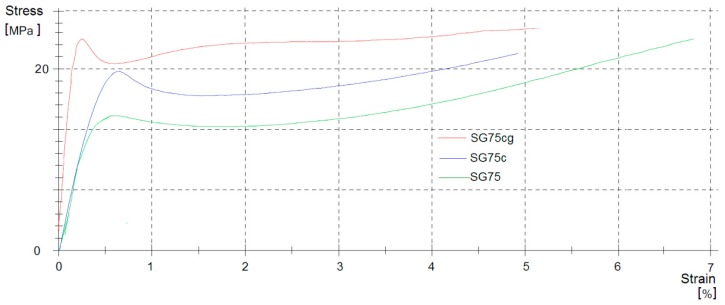
Representative strain curves from deformation (SG75, SG75c, SG75cg) in mechanical tests.

The composites produced by coating the agro-textile material with starch films were further analyzed in terms of their mechanical performance. Their tensile strength, elongation at break and hardness are given in Table 3 and in Figure 8. As can be seen, reducing the glycerin content caused a significant improvement in the mechanical properties of the composites. This was due to the chemical character of glycerin, which acts as a plasticizer in polymer compounds. The elongation at break of the composites gradually increased as the content of glycerin rose from 6.3% to 6.8%. The addition of glycerin to a polymer matrix enables the polymer chain molecules to bend and slide past each other more easily, resulting in greater flexibility.

The mechanical properties of all the cellulose-starch composites were significantly improved by the addition of the modifiers, casein or gelatin, while the difference in elongation at break was negligible. Gelatin caused a much greater improvement in mechanical properties. The increase in the mechanical properties of the SGg (SG75g, SG80g, SG85g) composites may be attributed to the level of organization in the protein network [39,41]. Casein is known to be a non-ordinate protein with a low level of α-helical and b-sheet structural conformations. As a consequence, the matrix of the films modified by casein was less organized than that of the gelatin films. The structure of gelatin is also less organized, but it is able to denature during the process of film production. Gelatin may also contain some parts of collagen, which has a higher degree of organization. The increase in the organization of the chain could improve the packing of molecules, which leads to the improvement of mechanical properties.

As can be seen in Table 3, reducing the glycerin content improved the hardness of the composites considerably, while they also showed less flexibility. This is because glycerin acts as a plasticizer. Glycerin was added in different percentage shares, i.e., 25% (SG75), 20% (SG80) and 15% (SG85). The smaller the proportion of glycerin in the composites, the greater was their hardness in degrees Shore (°Sh) and the lower was their flexibility. A decrease in hardness of up to 12% was observed for casein composites (SG75c) with a reduced percentage of glycerin. This was due to the different chemical structures of the modifiers.

#### 3.2.4. Thermo-Oxidative Aging Resistance

**Figure 9:**
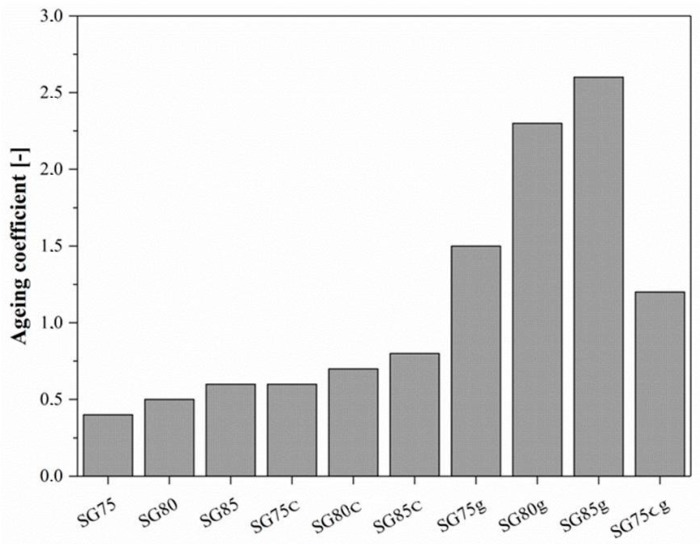
Effect of starch and glycerin content and of the addition of casein or gelatin as modifiers on the thermo-oxidative aging resistance of starch-based composites.

We also studied the effect of starch and glycerin content as well as of the addition of casein or gelatin as modifiers on the thermo-oxidative aging resistance of the starch-based composites. The ageing coefficient values for the composites are presented in Figure 9. As can be seen, the thermal aging resistance of the composites improved with increasing starch content. The improved mechanical properties after thermo-oxidative aging could be associated with the increase in cross-link density. The highest ageing coefficient (A) value were obtained for sample SG85g, whereas the composites containing gelatin exhibited high thermo-oxidative ageing progress.

#### 3.2.5. Microscopic Analysis

**Figure 10:**
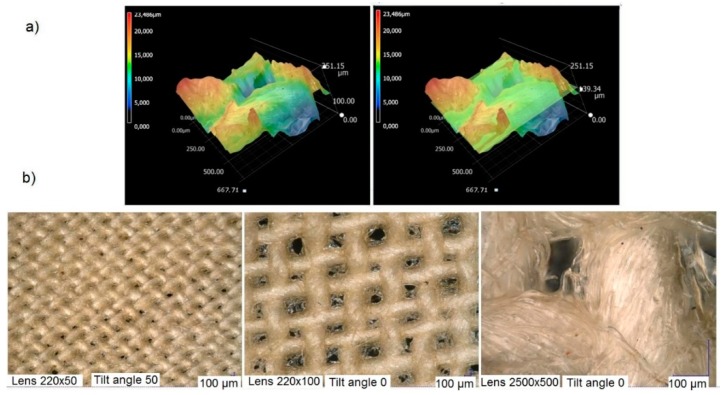
Three-dimensional 3D profile of Cellulose-starch composite (**a**); surface topographies at three magnifications (220 × 50; 220 × 100; 2500 × 500) (**b**). The images were made using a Keyence Microscope.

**Figure 11:**
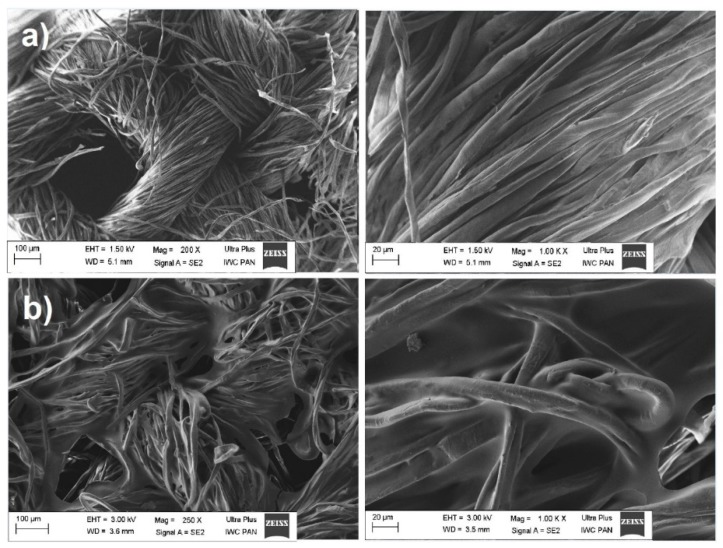
Pictures of SEM agro-fabrics uncoated (**a**) and coated with starch film (**b**), magnification 250× and 1000×.

Surface analysis of the cellulose-starch composites was made using a confocal microscope with high accuracy and resolution, and also by SEM (Figure 10 and Figure 11). Figure 10a shows images scanned in three-dimensional profile and Figure 10b images made at three magnifications.

The application of the starch coating on the cellulose fabric was uniform. This is clearly visible in the images made with both the 220 × 50 and 2500 × 500 lenses (Figure 10). In a 1000 μm wide section, cellulose fibers with a translucent glossy coating are visible. Free spaces not covered by the starch coating are noticeable over the space of the warp and weft. The 3D profile images show the nodes/values of the nanometer rows, which were measured based on the intensity of the reflected laser light in relation to the position of the Z axis of the laser. Depending on the clustering of the relevant types of molecules and the interactions between them, the color (fluorescent light reflected from the given surface) changes as a result of fluorescence resonance energy transfer. On the other hand, particle fluorescence is itself characterized by emission intensity, the emission spectrum or emission duration (so-called fluorescence lifetime). Deposition of the coating on the cellulose fibers resulted in the emission of red light, as seen in the 3D images, and the lack of clusters is the effect of the spaces not covered by starch suspension.

Similar observations can be made based on the SEM photos. Figure 11a shows the fibers of the non-coated fabric with a starch film at a magnification of 250× and 1000×. In contrast, in Figure 11b the structure of the plant is different, uniformly covered with a starch film, which contains the nutrients necessary for the growth of plants on this substrate. The fertilizing components contained in the starch films take longer to be released from the surface of the material and remain longer in the substrate, thus ensuring greater water absorption and more favorable conditions for cultivated plants during periods of drought.

#### 3.2.6. Thermal Analysis DTA, DSC

**Figure 12:**
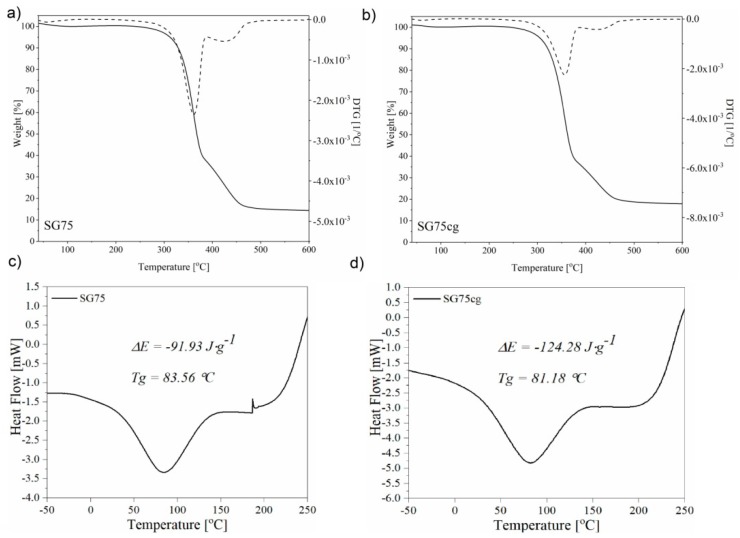
Effect of keratin modification on TG-DTG starch composites: SG75 (**a**), SG75cg (**b**). Effect of casein and gelatin biopolymers on DSC of starch composites: SG75 (**c**), SG75cg (**d**).

The thermal analysis of the samples in the scraper composites shows that the 50% T_50_ temperature loss of the sample mass is at a similar level in the case of degradation for the sample containing casein and gelatin biopolymers as in the SG75 composition (Figure 12).

Therefore, the total loss of sample mass is lower for starch composites containing biopolymers, which means that there is a larger thermal order residue in the cases of the casein and gelatin compositions. It can also be seen that SG75 and SG75cg are differently distributed, while a one-stage distribution is visible for SG75 on the TG curve. The disintegration of SG75cg proceeds clearly in three stages (Figure 12). The compositions disintegrate with large weight loss. This distribution begins already starts at about 150 °C and in the case of SG75cg decomposition occurs from 200 °C. For SG75cg, three change domains can be seen on the thermal curves, occurring in the temperature ranges of about 300–400, 400–500 and 600–650 °C. The first change indicates loss of mass and can be attributed to the degradation of the composition substrate, which is the fabric. The second change may be the result of the degradation of the starch-ducal-gelatin layer. The third is associated with a significant loss of mass. The DSC analysis shows that the flexibility of the SG75cg samples decreased slightly, as indicated by the increased glass transition temperature Tg. This is consistent with the observed mechanical properties. The SG75cg material is more rigid, and thus exhibits higher hardness values. This may result from interaction between the starch matrix and biopolymers, which reduces segmental mobility in the phase contact, which in turn causes a change in the Tg value at higher temperatures, as shown by the DSC analysis. The DSC curves for the starch composites exhibit wide endothermic peaks at 83.6, 86.9 and 81.2 °C, respectively, for SG75 and SG75cg. This may be the effect of water loss due to the breakage of bonds with the cellulose fabric. Therefore, the initial temperature of the decomposition started from about 25 °C, the peak is observed at around 83 °C and the end temperature is about 132 °C. Two Tg can be observed for the composites: A distinct difference in the heat of combustion Cp is visible at a temperature of around −10 °C and a more subtle one at around 72 °C. The noticeably larger residue after the thermal decomposition of the SG75cg material, its higher stiffness, mechanical strength and thus increased Tg values, may cause this composite to have desirable properties as a substrate for growing plants.

#### 3.2.7. Implantation of Beans on Cellulose-Starch Composites and Composting Tests

**Figure 13:**
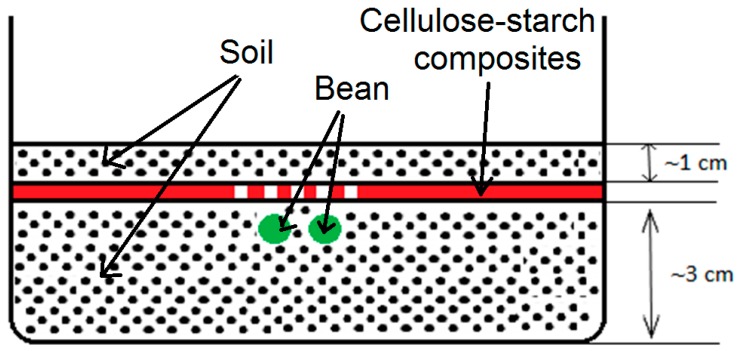
Scheme showing the sowing of legumes (*Phaseolus vulgaris*) on cellulose-starch composites.

Controlled composting is a reliable test method also for starch and cellulose, and consequently for starch and cellulose based materials. Composites made of these materials are not only biodegradable but also have good mechanical properties, as shown by numerous scientific studies [41,42,43,44]. Figure 13 gives a schematic view of the sowing of bean-type crops (Phaseolus vulgaris) on the cellulose-starch composites. Over a two-week period, observations were made to monitor how the legume grew in comparison to beans sown in a container to which the composite had not been applied. The tests and all observations were carried out at room temperature.

**Figure 14:**
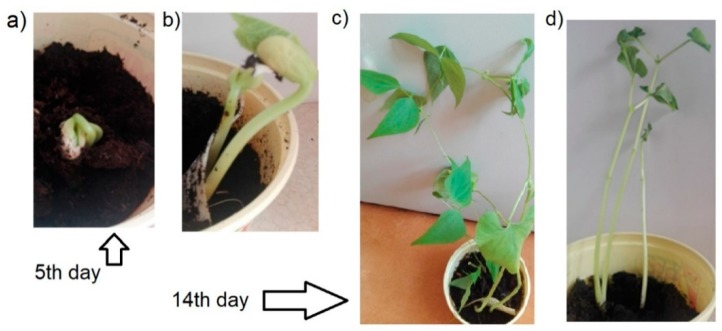
Growth of legume beans after 5 (**a**,**b**) and 14 (**c**,**d**) days from sowing.

Figure 14a,c shows the growth of plants without the use of any composite and Figure 14b,d with composites. Five days after sowing, a small nucleus could be seen piercing through the soil without composite (Figure 14a), while the use of cellulose-starch composites resulted in a significant increase in the size of the germinating plant (Figure 14b). After two weeks, the bean stalks reached a height of 12–20 cm in the absence of a starch cover and of 25–32 cm with the use of the cellulose-starch composites. The substrate, in the form of natural biopolymers (i.e., starch, casein or gelatin) contained nutrients and mineral supports for the growth of crop plants.

**Figure 15:**
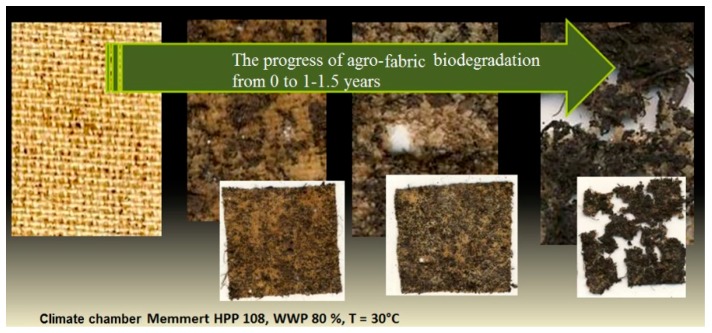
Progress of biodegradation the starch-cellulosic composite from 0 to 1–1.5 years.

Subsequent studies included composting of the starch-cellulosic composite for the assessment of biodegradability (Figure 15).

As a result of the tests, degradation in 80% of the starch-cellulosic composite and significant defragmentation were estimated over an annual/half year period. Under the influence of hydrolytic factors in the environment, the first layer of the coating composition decomposes, followed by the subsequent disintegration of the warp and weft of the strands of the cellulose iron and then by total looseness of cohesion. As the degradation progresses, soil adhesion to the material increases, as a result of the mechanism of mechanical degradation. After a period of surface use, i.e., about 1–1.5 years, the agro-textile material may be completely biodegraded without requiring costly removal and further composting.

## 4. Conclusions

The results of this study confirm that the addition of glycoproteins, phosphoproteins and long polypeptide chains to a starch matrix increases the interactions between the RCOO and NHCO groups in both films and cellulosic composites. Starch films and starchy compositions were prepared based on the modification of potato starch biopolymers with similar properties and constructions. Different proportions of glycerin, casein or gelatin were used to modify the composites, as well as a medium made of cellulosic material to reinforce the material. Both the introduction of casein and gelatin improved the structure of starch films, as confirmed by infrared spectral analysis as well as surface pictures made by scanning electron microscopy (SEM). Differences were observed in the intensity of absorption bands from the groups –C=O, –OR and –OH. Increasing hardness values and sharper increases in water absorption during equilibrium swelling were also noted with respect to the native sample. The application of the films to natural cotton led to the formation of a stable structure between the gelatin or casein starch material and the cellulose base, as confirmed by confocal microscope analysis and SEM. This resulted in improved crosslink density, as shown by infrared spectral analysis, as well as more favorable mechanical properties. The starch composites produced, as shown in the thermal analysis of DTA, DSC, together with the addition of casein and gelatin, are characterized by a higher glass transition temperature Tg, greater rigidity, which is confirmed by the mechanical tests, hardness. The material should be stable for atmospheric conditions when used in crops. Similar observations have been made for cellulose-elastomeric material by Prochon and Tshela Ntumba [45]. Starch films and starchy compositions on cellulose fabrics are environmentally friendly materials, which undergo composting and biodegradation, with the potential to be used as agro-fabrics for the cultivation of crop plants.

## Figures and Tables

**Table 1:** Composition of starch films with gelatin or casein as a modifiers.

Component	Quantities of Ingredients (phr)									
	SG75	SG80	SG85	SG75c	SG80c	SG85c	SG75g	SG80g	SG85g	SG75cg
Starch (S)	75	80	85	75	80	85	75	80	85	75
Glycerin (G)	25	20	15	20	15	10	20	15	10	15
Casein (c)	0	0	0	5	5	5	0	0	0	5
Gelatin (g)	0	0	0	0	0	0	5	5	5	5

SG75—film with starch (75 phr), glycerin (25 phr); SG80—film with starch (80 phr), glycerin (20 phr); SG85—film with starch (85 phr), glycerin (15 phr); SG75c—film with starch (75 phr), glycerin (20 phr), casein (5 phr); SG80c—film with starch (80 phr), glycerin (15 phr), casein (5 phr); SG85c—film with starch (85 phr), glycerin (10 phr), casein (5 phr); SG75g—film with starch (75 phr), glycerin (20 phr), gelatin (5 phr); SG80g—film with starch (80 phr), glycerin (15 phr), gelatin (5 phr); SG85g—film with starch (85 phr), glycerin (10 phr), gelatin (5 phr); SG75cg—film with starch (75 phr), glycerin (15 phr), casein (5 phr), gelatin (5 phr).

**Table 2:** Hardness and equilibrium swelling in water (Qw) of selected starch films, and color change coefficient (dE*ab) and hardness of starch films after biodegradation (∆ Hardness).

Compound	Hardness [^o^Sh]	∆ Hardness [%]	Qw [-]	dE*ab [-]
SG75	49.0 ± 0.5	8.98 ± 0.11	1.38 ± 0.02	2.59 ± 0.02
SG80	55.8 ± 0.1	5.87 ± 0.23	1.28 ± 0.04	1.59 ± 0.03
SG85	77.6 ± 0.3	5.80 ± 0.06	1.37 ± 0.05	2.84 ± 0.05
SG75g	65.9 ± 0.7	8.63 ± 0.08	1.51 ± 0.01	2.27 ± 0.04
SG80g	62.1 ± 0.4	6.67 ± 0.10	1.41 ± 0.01	2.33 ± 0.04
SG85g	60.3 ± 0.3	7.02 ± 0.08	1.34 ± 0.02	1.55 ± 0.02
SG75c	65.9 ± 0.7	8.96 ± 0.08	1.40 ± 0.02	3.77 ± 0.05
SG80c	77.2 ± 0.8	11.31 ± 0.08	1.27 ± 0.06	2.39 ± 0.03
SG85c	78.2 ± 0.4	9.68 ± 0.08	1.28 ± 0.02	1.68 ± 0.02
SG75cg	54.8 ± 0.3	6.02 ± 0.09	1.42 ± 0.01	3.22 ± 0.02

**Table 3:** Effect of the impact of starch and glycerin content and of the addition of casein or gelatin on the mechanical properties of cellulose-starch composites.

COMPOSITE		Tensile Strength (MPa)	Elongation at Break (%)	Hardness (°Sh)
SG75	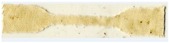	20.1 ± 1.3	6.8 ± 0.1	49.6 ± 0.3
SG80	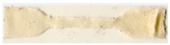	22.2 ± 1.1	6.5 ± 0.2	51.2 ± 0.3
SG85	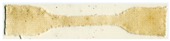	24.2 ± 1.5	6.3 ± 0.1	52.7 ± 0.3
SG75c	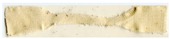	23.3 ± 1.3	4.8 ± 0.3	70.5 ± 0.3
SG80c	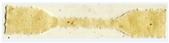	25.9 ± 1.4	4.7 ± 0.3	76.7 ± 0.3
SG85c	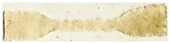	28.3 ± 1.7	4.4 ± 0.3	78.6 ± 0.3
SG75g	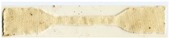	23.6 ± 1.2	6.3 ± 0.3	59.1 ± 0.3
SG80g	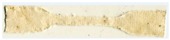	32.1 ± 1.8	6.1 ± 0.3	62.1 ± 0.3
SG85g	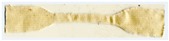	33.2 ± 1.7	5.9 ± 0.3	64.3 ± 0.3
SG75Cg	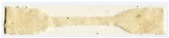	25.5 ± 1.3	5.9 ± 0.3	65.4 ± 0.3
